# Increased Host Species Diversity and Decreased Prevalence of Sin Nombre Virus

**DOI:** 10.3201/eid1507.081083

**Published:** 2009-07

**Authors:** Laurie J. Dizney, Luis A. Ruedas

**Affiliations:** Portland State University, Portland, Oregon, USA

**Keywords:** Sin Nombre virus, hantavirus, species diversity, zoonoses, viruses, research

## Abstract

Emerging outbreaks of zoonotic diseases are affecting humans at an alarming rate**.** Until the ecological factors associated with zoonoses are better understood, disease emergence will continue. For Lyme disease, disease suppression has been demonstrated by a dilution effect, whereby increasing species diversity decreases disease prevalence in host populations. To test the dilution effect in another disease, we examined 17 ecological variables associated with prevalence of the directly transmitted Sin Nombre virus (genus *Hantavirus*, etiologic agent of hantavirus pulmonary syndrome) in its wildlife host, the deer mouse (*Peromyscus maniculatus*). Only species diversity was statistically linked to infection prevalence: as species diversity decreased, infection prevalence increased. The increase was moderate, but prevalence increased exponentially at low levels of diversity, a phenomenon described as zoonotic release. The results suggest that species diversity affects disease emergence.

## Abstract 2

Prevalence of infection was highest where fewer animal species carried the virus.

During the past 60 years, the number of emerging pathogens affecting humans has substantially increased (*1*). Of these emerging infectious diseases, 62% are zoonotic (*2*), meaning they are naturally hosted by, and persist in, wildlife but also affect human populations. The ecological factors associated with zoonotic disease emergence are likely complex and are poorly understood. Most often, because of limited time, resources, and the exigencies of the situation, outbreak investigations of emerging diseases seek only to discover the pathogen responsible for the disease in humans. But ecological studies are of critical importance to long-term containment of zoonotic disease emergence; they are the only way to ascertain the wildlife source of the disease, the dynamics of the host–pathogen relationship, and the ecological factors associated with an outbreak. Knowledge of all these factors is needed to proactively protect the public from zoonotic diseases; without this knowledge, new diseases will continue to emerge. The worldwide distribution of these largely zoonotic diseases suggests a globally distributed mechanism for their emergence.

Anthropogenic factors—including pollution, land-use conversions, and climate change—likely contribute to disease emergence by several mechanisms (*3*), one of which has been hypothesized to be decreased species diversity. The number of species currently being lost, as well as the rate of species loss, is unprecedented (*4*); these losses generally have negative effects on ecosystem functioning (*5*,*6*). It likely is not coincidental that areas where many zoonoses are emerging among humans are the same areas where loss of species is accelerating, e.g., Central Africa (Ebola, monkeypox, Marburg virus), West Africa (Lassa virus, HIV-2), Southeast Asia (Nipah virus, severe acute respiratory syndrome, avian influenza), and South America (dozens of strains of hantaviruses and arenaviruses).

Lyme disease, a vector-borne zoonosis, is affected by loss of species by a process known as the dilution effect (*7*), whereby increasing species diversity decreases disease prevalence by diluting the availability of competent hosts with increased numbers of noncompetent hosts. Little research on the dilution effect has been carried out beyond its effect on Lyme disease (*8*), yet the global implications of the phenomenon—if the effects are applicable to other types of diseases and transmission dynamics—could have substantial and enduring effects on human health and conservation.

Hantaviruses provide a model system in which to test the dilution effect in directly transmitted zoonoses. Since their initial discovery in the Western Hemisphere in 1982, several dozen hantavirus strains have been found, each hosted by a unique rodent species (*9*); novel hantaviruses have recently been discovered in shrews (*10*,*11*). Natural hosts are asymptomatic and chronically infected; intraspecies spread is hypothesized to be through bites (*12*). Humans become infected with hantavirus by inhaling aerosolized excreta from infected rodents (*13*). Occasionally hantavirus pulmonary syndrome (*14*) develops; this syndrome has a mortality rate of almost 40% and no prophylaxis, treatment, or cure (*15*). Most of the 506 confirmed cases in the United States have been caused by Sin Nombre virus (SNV). Studies have found that low diversity ecosystems dominated by the rodent hosts for 3 distinct hantaviruses had high infection prevalence in the host (*16*,*17*), suggesting a role for species diversity. Although the mechanism of disease dilution would differ in directly transmitted zoonoses (e.g., hantaviruses), as opposed to vector-borne diseases, a dilution effect could occur if 1) individuals of the host species remain as species diversity decreases, 2) the disease is spread within the host species through direct encounters (such as biting), and 3) presence of other species causes encounters among the host species to decrease.

Other ecological factors could affect the number of intraspecific deer mouse (*Peromyscus maniculatus*) encounters, including increased density of deer mice and vegetative factors that lead to variation in population numbers (e.g., available cover and forage) (Table 1). Some studies have found high SNV prevalence in host populations when deer mice densities were high (*18*,*19*). However, although the concept of density-dependent transmission is not unique to hantaviruses, its applicability to the deer mouse–SNV system has been elusive. SNV prevalence also has been shown to vary with habitat characteristics and quality (*15*,*18*,*19*), although interpretation of this variation has been difficult because SNV prevalence varies as much within as among habitat types (*20*).

**Table 1:** Vegetative factors measured within each site and their transformations, Portland, Oregon, USA, October 2002−September 2005

Habitat	Description	Transformation
Tree cover	% Plot covered with trees	log_10_ +1
Shrub cover	% Plot covered with shrubs	Square root
Bryophyte	% Plot covered with bryophytes	Square root
Bare ground	% Plot that is bare ground	log_10_ +1
Bare ground and litter	% Plot that is bare ground and bare ground covered with litter	Square root
Ground cover	% Ground of plot that has any cover, including plants, logs, litter	None
Plant ground cover	% Plot that has only plant ground cover	None
Coarse woody debris	% Plot that is logs, stumps, snags	log_10_ +1
Trees	No. all trees	log_10_ +1
Large trees	No. trees >25 cm circumference	log_10_ +1
Maximum tree height	Tallest tree in plot	None
Total shrubs	No. all shrubs	log_10_ +1
Small shrubs	No. shrubs <99 cm tall	Omitted
Large shrubs	No. shrubs >100 cm tall	Omitted
Plant species	No. plant species	None

In this study we examined small mammal populations in 5 forested sites over a 3-year period, October 2002 through September 2005. We monitored mammal species diversity, deer mouse densities, and SNV infection prevalence in the mammals to test the hypotheses that 1) areas of higher mammal species diversity would exhibit lower prevalence of SNV infection in host populations, 2) areas of higher host density would contain higher infection prevalence of SNV in the host populations, and 3) vegetative factors could be related to prevalence of SNV infection among deer mice.

## Materials and Methods

### Sites

We sampled small mammals at 5 sites in and around Portland, Oregon, USA: site 1, Forest Park (45.5916°N, 122.7983°W); site 2, Tryon Creek State Park (45.4337°N, 122.6690°W); site 3, Powell Butte Portland City Park (45.4837°N, 122.5059°W); site 4, Oxbow Metro Regional Park (45.4879°N, 122.2970°W); and site 5, Tualatin River National Wildlife Refuge (45.3957°N, 122.8305°W). Detailed site descriptions can be found in Dizney et al (*21*).

### Trapping and Blood Sampling

To sample as many different mammal species as possible, we set up a trapping web 200 m in diameter (*22*) at each site and used 4 trap types: Sherman (H.B. Sherman Traps, Tallahassee, FL, USA), handmade wire mesh, Tomahawk (Tomahawk Live Trap Co., Tomahawk, WI, USA), and pitfall. Each station included an aluminum folding Sherman live trap and a custom-built mesh live trap (*23*) of similar dimensions (7.6 cm × 8.9 cm × 22.9 cm). Two sizes of Tomahawk live traps were used to trap larger animals; a 61 cm × 17.8 cm × 17.8 cm trap was placed at each 50-m trap station, and a 91.4 cm × 25.4 cm × 30.5 cm trap was placed at each 100-m trap station. Pitfall traps were made by using a 19-L bucket (30-cm diameter, 36-cm depth) with a lid for rain and predator cover suspended ≈8 cm above ground to enable access by small animals (*24*); pitfall traps were placed at each 20-, 50-, and 100-m trap station. The center point of each trapping web contained 2 Sherman and 2 mesh traps at 90° angles to each other. The total number of traps in each sampling grid was 352. Each park was trapped 19 times (4 nights each time) from October 2002 through September 2005, approximately every 8 weeks. Traps were checked each morning. The sampling was specifically designed such that densities, diversities, and infection prevalence could be compared across space and time. The total trap effort (traps × nights) was 133,760 trap-nights. Sherman and mesh traps were baited with a mixture of peanut butter and rolled oats, Tomahawk traps were baited with cat food, and pitfall traps were not baited. To reduce deaths from hypothermia, we added polyfiber nesting material to Sherman and mesh traps when warranted by the weather.

All captured animals were treated as if they were infected with SNV, and standard precautionary methods were implemented (*25*). After point of capture was recorded, animals were transferred from traps to sealable plastic bags or, if too large, left in the trap and brought to the center of the web, where they were weighed and measured and examined for age, sex, reproductive status, scarring, or other notable characteristics. Retroorbital blood samples were collected by using heparinized microcapillary tubes and either placed in cryovials and frozen in liquid nitrogen or placed in serum separator tubes and refrigerated for no more than 1 week before testing. Infection prevalence was determined by ELISA (*26*). Infected deer mice were counted 1 time (time of first capture).

During the first 2 years of the study, to obtain tissue samples for a companion study, deer mice were euthanized in a chloroform chamber (*25*). The resulting specimens were tagged and stored at the Museum of Vertebrate Biology at Portland State University. All other animals captured were marked with ear tags and released at the point of capture. During the last year of the study, deer mice were also tagged and released. To determine whether removal affected subsequent capture rates within the same trapping period, the differences between the number of captures on the first and last day of the trapping period were calculated and averaged, then compared between removal and replacement sampling with the Welch 2-sample *t*–test. Because no significant differences were found between the first 2 years and the last year of the study (*t* = 0.50, p = 0.63, df = 8), data from all 3 years were analyzed together. This research was conducted under the auspices of federal, state, and city permits, and it complied with the American Society of Mammalogists’ guidelines for animal care and use (*27*).

### Species Diversity and Density

Deer mouse density was calculated by using the Distance program (*28*). Mammal species diversity was measured by using the Simpson diversity index (D_S_) (*29*), which takes into account both richness (number of species) and evenness (number of individuals within each species) and ranges from 0 (least diversity) to 1 (maximal diversity). D_S_ further represents the probability of interspecies encounters (*30*). Pairwise comparisons of D_S_ values among parks was conducted by using the Student *t* test; differences of D_S_ were divided by the square root of their variances (*30*). To minimize the possibility of type 2 errors resulting from multiple comparisons, a statistically conservative Bonferroni correction was made (α = 0.05/10 comparisons, or 0.005) (*31*). Deer mouse densities were compared pairwise by using the Welch 2-sample *t* test. Logistic regression with binomial errors was initially used to assess the association between infection prevalence and deer mouse density and species diversity. However, the resulting models showed such extensive overdispersion that we considered logistic regression to be an unsuitable statistical method for these data (*32*). Accordingly, we used nonlinear regression analysis.

### Analysis of Similarity

An analysis of similarity returns a statistic (R) based on a Bray–Curtis dissimilarity measure, which considers the difference of the mean ranks between and within groups. Most values fall between 0 and 1; 1 is the most dissimilar. Significance is assessed by comparing the observed value of R to the permutation distribution of R (*33*). Again, because of multiple comparisons, a Bonferroni correction was made such that α = 0.005 (*31*). We then used stepwise (backward) logistic regression with binomial errors to assess the association between infection prevalence and vegetative characteristics.

## Results

Although only 5 sites were examined, the intensity of the sampling yielded a total of 5,057 individuals from 21 species, resulting in a thorough species inventory over a gradient of diversity in small mammal ecological communities. Deer mice averaged 62% of all captures (Table 2) and were the dominant species at all sites. Mammal species diversity differed significantly among sites (p<0.001; Table 2), except sites 3 and 4 (p = 0.1). Densities varied spatially and temporally; all parks exhibited the highest densities during year 2 (Table 3). Interannual variances of densities were large due to seasonal differences in capture rates, such that no statistical differences in densities were found either within or among parks. Infection prevalence also varied, although it remained consistently low at 4 of the 5 sites. During year 1, infection prevalence was significantly higher at site 1 than at sites 2 and 3 (p<0.001) but not different than at sites 4 and 5 (p = 0.20 and 0.32, respectively). Site 1 was the only site where infection prevalence significantly increased between years 1 and 2 (p = 0.005); thus, infection prevalence at this site was significantly higher than at any of the other parks during year 2 (p<0.001). High infection prevalence was maintained at site 1 during year 3. Although the rate for site 2 increased significantly between years 2 and 3 (p = 0.035), prevalence remained significantly higher at site 1 than at any other park during year 3 (p<0.01).

**Table 2:** Small mammal capture data for 5 parks, Portland, Oregon, USA, October 2002−September 2005

Site no.	Total no. mammals captured	No. deer mice (*Peromyscus maniculatus*) captured	No. deer mice/total no. captured	No. species	*Simpson diversity index, D_S_*
1	1,032	798	0.773	12	*0.385*
2	1,248	884	0.708	11	*0.461*
3	730	492	0.674	11	0.532
4	862	862	0.633	16	0.560
5	1,185	472	0.398	16	0.753

**Table 3:** *Comparison of deer mouse (*Peromyscus maniculatus*) density and Sin Nombre virus infection prevalence, Portland, Oregon, USA, October 2002−September 2005*

Site no.	Deer mouse density				*Infection prevalence*		
	Year 1	Year 2	Year 3		Year 1	Year 2	*Year 3*
1	6.78	22.38	8.76		0.049	0.141*	*0.148*
2	13.77	32.86	24.71		0.004†	0.011†	0.037*†
3	8.57	13.62	8.11		0.000†	0.013†	*0.044†*
4	15.92	23.34	7.30		0.015	0.004†	*0.030†*
5	7.74	23.43	7.80		0.021	0.012†	*0.023†*

*Significance between years at α = 0.05 using a test of homogeneity of proportions with the Yates continuity correction. *†Significance between site 1 and other sites at α = 0.05 with the Fisher exact test for count data.*

Using nonlinear regression, we found a significant negative relationship between infection prevalence and mammal species diversity. Infection prevalence increased as diversity decreased, up to an inflection point where the rate of infection increased exponentially (Figure). No regression model was able to account for the association between infection prevalence and density of deer mice, either alone or with species diversity in the model.

**Figure:**
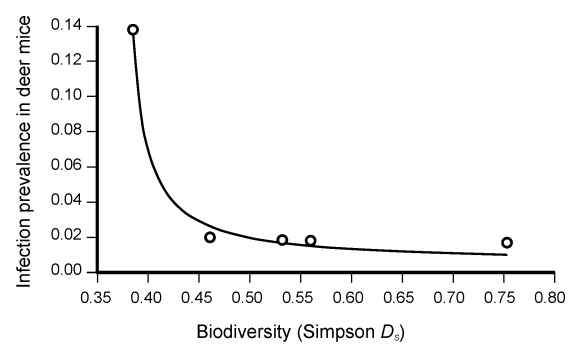
Results of the nonlinear regression analysis between species diversity (expressed as Simpson diversity index, *D_s_*) and Sin Nombre virus prevalence among deer mice (*Peromyscus maniculatus*) at each of 5 parks in Portland, Oregon, USA. The best fit model was of the form Y = x / (ax + b), *R^2^* of 0.9994, p = 0.00001. The figure represents a summary of the results in that it shows the averages of all the seasons, in all years, in each park (indicated by circles). A regression using individual seasons and parks shows the same results.

A pairwise analysis of similarity was used to compare sites floristically; all parks differed significantly from each other (p<0.001). Stepwise backward logistic regression with binomial errors found no association between infection prevalence and any vegetative factors alone or in combination with other vegetative factors.

## Discussion

Population densities fluctuated synchronously at all sites, yet infection prevalence increased significantly at only 1 site, which suggests that factors other than density alone are involved in disease transmission. If, as hypothesized, transmission were through aggressive encounters (*12*), SNV would spread most efficiently in an ecosystem composed solely of deer mice, where every encounter would be a potential disease-transmitting encounter. As more species, and more individuals within those species, are added to the community, the number of potential disease-transmitting encounters decreases because species other than deer mice are nonhost (not competent, or nonamplifying) species. This type of decreased intraspecies interaction has been termed “encounter reduction” (*34*) and would occur if increasing species diversity increases the number of competitors in an ecosystem, thereby increasing the amount of time a host species has to spend securing limited resources (food, nest sites), in turn decreasing the time spent on intraspecies encounters.

An increase in species diversity, in combination with an increase in the densities of individuals within those species, as we observed in this study, should also mean an increase in the number of predators of the rodent host species. It is reasonable to hypothesize that predators keep rodent numbers under control, in turn limiting pathogen spread both among rodents and into human populations, although it has been difficult to empirically support this hypothesis (*35*). Our results suggest that predators control infection prevalence not by controlling the density of host species but instead by an unrelated mechanism, possibly encounter reduction. When predators are present in the ecosystem, host species should spend more time in the nest, in hiding, or within the familiarity of their territory, all to avoid predation and all likely to decrease intraspecies encounters. This hypothesis is supported by the fact that capture rate—but not density—was highest at site 1 during year 2 relative to all other parks (p<0.01), which means that deer mice were moving about and encountering traps more often. We hypothesize that when predation and competition are decreased or absent, for this small mammal community at a Simpson diversity index ≈0.43, a zoonotic release of predatory and competitive controls appears to have occurred, in which SNV infection prevalence increased drastically. This hypothesis would account for the lack of differences in infection prevalence rates at sites 2–5; although the Simpson index for these sites varied significantly; the threshold for zoonotic release had not been breached at any of those sites. Above the threshold level, sites would maintain a low level of infection, or perhaps locally lose infection altogether. In this study, SNV infection prevalence was either so low during some seasons at some sites as to be virtually undetectable by traditional trapping techniques or ephemerally absent. In particular, SNV was undetected or absent most often at the most diverse site (no SNV was detected in 8 of 12 seasons at site 5, in 6–7 seasons at sites 2–4, and in 1 season at site 1). Our system differs from the Lyme disease system, which depends on a vector that is not host specific (black-legged tick) to transmit the disease. Here, in contrast, presence of nonhost species in the small mammal community will not directly affect the transmission of SNV; instead, the behavior of members of the natural host species will be affected, decreasing SNV transmission rates among competent hosts through encounter reduction. Increased diversity in both the Lyme disease and SNV systems appears to lead to decreased disease prevalence, although the mechanisms differ. Another difference between the 2 systems is the threshold relationship between species diversity and SNV prevalence, which suggests that the shape of the dilution curve may be mechanism dependent and is the reason we proposed the term “zoonotic release.” Given that many hantaviruses are hosted by generalist rodent species (i.e., those able to exploit a broad variety of ecological resources) that dominate ecosystems as species diversity decreases (e.g., Laguna Negra virus in vesper mice [*Calomys* spp.]; Andes and Choclo viruses in colilargos [*Oligoryzomys* spp.]; and Calabazo virus in cane rats [*Zygodontomys* spp.]), this type of zoonotic release could be widespread throughout the host–virus system in the genus *Hantavirus*.

Host density should likewise be considered a factor in this phenomenon because density increased before infection prevalence increased. However, the result of the logistic regression between density and infection prevalence, although significant, was marked by considerable overdispersion, suggesting that this was the wrong model, and its significance was greatly overestimated (*32*). Additionally, at all parks deer mouse density increased but infection prevalence did not, clearly indicating that density is not the sole driver of infection prevalence in this system. A logistic regression with both density and mammal species diversity in the model showed similar overdispersion. Our results suggest that dependence on both density and frequency play a role in SNV transmission, which may be one of the reasons it has been so hard to determine their respective roles in the transmission of hantaviruses (*36*). More extensive studies should therefore be undertaken wherein species diversity, density, and frequency of encounters are carefully measured to determine their respective roles in disease transmission.

The finding that infection prevalence of a directly transmitted zoonosis may be inversely related to species diversity has implications for human health. The toll in illness and death from emerging zoonotic diseases is high, and outbreak investigations are costly (*37*). These investigations often fail to identify the source of a pathogen, let alone answer the question of why an outbreak occurred at a given time and place. If the host species or vector is found, eradication usually is neither possible nor desirable, particularly when the species are as ubiquitous as deer mice. Prophylaxis is difficult when transmission is airborne, as in hantaviruses, for which potentially everyone in a region is at risk. Ecosystem-level control may be the best way to protect the public from the increasing threat of many zoonotic diseases. Wildlife also are at risk for infection with novel pathogens, and the factors underlying wildlife disease emergence are similar to those in humans (*38*); a dilution effect may therefore help protect wildlife as well. For example, a study of West Nile virus suggested that increased bird species richness depressed the prevalence of the virus in ecosystems (*39*). Thus, wildlife could be protected in 2 ways: first, from dilution of diseases that are potentially harmful to them and second, from maintenance of healthy ecosystems.

Extension of a dilution effect to directly transmitted diseases has implications for conservation as well. Although protecting species diversity is a cause that would seem universal in its appeal, conservationists often are perceived as being overly biocentric and having little concern for human welfare. In addition, many benefits derived from maintaining diverse ecosystems are difficult for the layperson to decode and seem far removed from daily life such that despite scientific research, unparalleled loss of species caused by anthropogenic factors continues at an unabated rate. Conservation likely will not succeed without the support of the general public, who in turn influence the environmental policies our society embraces. To gain support of the general public, tangible human benefits from conservation should outweigh the immediate—usually economic—gains of nonconservation land use (*40*). Linking human health to biodiversity could be just the benefit for gaining the public’s support of conserving biodiverse ecosystems. Protection from disease is a tangible objective; it is easily understood and translated and it has direct benefits for all. As a consequence, extension of a dilution effect to directly transmitted diseases could have broad conservation implications by raising the public’s concern about conservation in a manner that has yet to be emphasized.

## References

[1] Jones KE, Patel NG, Levy MA, Storeygard A, Balk D, Gittleman DJ, Global trends in emerging infectious diseases. Nature. 2008;451:990–3. 10.1038/nature06536 18288193 PMC5960580

[2] Taylor LH, Latham SM, Woolhouse ME. Risk factors for human disease emergence. Philos Trans R Soc Lond B Biol Sci. 2001;356:983–9. 10.1098/rstb.2001.0975 11516376 PMC1088493

[3] Sutherst RW. Global change and human vulnerability to vector-borne diseases. Clin Microbiol Rev. 2004;17:136–73. 10.1128/CMR.17.1.136-173.2004 14726459 PMC321469

[4] Pimm SL, Raven P. Biodiversity: extinction by numbers. Nature. 2000;403:843–5. 10.1038/35002708 10706267

[5] Cardinale BJ, Palmer MA, Collins DJ. Species diversity enhances ecosystem functioning through interspecific facilitation. Nature. 2002;415:426–9. 10.1038/415426a 11807553

[6] Hector A, Bagchi R. Biodiversity and ecosystem multifunctionality. Nature. 2007;448:188–96. 10.1038/nature05947 17625564

[7] Ostfeld RS, Keesing F. The function of biodiversity in the ecology of vector-borne zoonotic disease. Can J Zool. 2000;78:2061–78. 10.1139/cjz-78-12-2061

[8] Dobson AP, Cattadori I, Holt RD, Ostfeld RS, Keesing F, Krichbaum K, Sacred cows and sympathetic squirrels: the importance of biological diversity to human health. PLoS Med. 2006;3:714–8. 10.1371/journal.pmed.0030231 PMC1472550 16729846

[9] Monroe MC, Morzunov SP, Johnson AM, Bowen MD, Artsob H, Yates TL, Genetic diversity and distribution of *Peromyscus*-borne hantaviruses in North America. Emerg Infect Dis. 1999;5:75–86. 10081674 10.3201/eid0501.990109 PMC2627704

[10] Klempa B, Fichet-Calvet E, LeCompte E, Auste B, Aniskin V, Barriere P, Novel hantavirus sequences in shrew, Guinea. Emerg Infect Dis. 2007;13:520–2. 17554814 10.3201/eid1303.061198 PMC2725914

[11] Arai S, Song JW, Sumibcay L, Bennett SN, Nerurkar VR, Parmenter C, Hantavirus in northern short-tailed shrew, United States. Emerg Infect Dis. 2007;13:1420–3. 18252128 10.3201/eid1309.070484 PMC2262104

[12] Mills JN, Yates TL, Ksiazek TG, Peters CJ, Childs JE. Long-term studies of hantavirus reservoir populations in the southwestern United States: rationale, potential, and methods. Emerg Infect Dis. 1999;5:95–101. 10081676 10.3201/eid0501.990111 PMC2627686

[13] Tsai TF. Hemorrhagic fever with renal syndrome—mode of transmission to humans. Lab Anim Sci. 1987;37:428–30. 2889846

[14] Duchin JS, Koster FT, Peters CJ. Hantavirus pulmonary syndrome: a clinical description of 17 patients with a newly recognized disease. N Engl J Med. 1994;330:949–55. 10.1056/NEJM199404073301401 8121458

[15] Yates TL, Mills JN, Parmenter CN, Ksiazek TG, Parmenter RR, Vande Castle JR, The ecology and evolutionary history of an emergent disease: hantavirus pulmonary syndrome. Bioscience. 2002;52:989–98. 10.1641/0006-3568(2002)052[0989:TEAEHO]2.0.CO;2

[16] Yahnke CJ, Meserve PL, Ksiazek TG, Mills JN. Patterns of infection with *Laguna Negra* virus in wild populations of *Calomys laucha* in the central Paraguayan Chaco. Am J Trop Med Hyg. 2001;65:768–76. 11791973 10.4269/ajtmh.2001.65.768

[17] Ruedas LA, Salazar-Bravo J, Tinnin DS, Caceres L, Garcia A, Eskew LJ, Community ecology of small mammal populations in Panamá following an outbreak of hantavirus pulmonary syndrome. J Vector Ecol. 2004;29:1–15. 15266755

[18] Mills JN, Ksiazek TG, Ellis BA, Rollin PE, Nichol ST, Yates TL, Patterns of association with host and habitat: antibody reactive with Sin Nombre virus in small mammals in the major biotic communities of the southwestern United States. Am J Trop Med Hyg. 1997;56:273–84. 9129529 10.4269/ajtmh.1997.56.273

[19] Biggs JR, Bennett KD, Mullen MA, Haarmann TK, Salidbury M, Robinson RJ, Relationship of ecological variables to Sin Nombre virus antibody seroprevalence in populations of deer mice. J Mammal. 2000;81:676–82. 10.1644/1545-1542(2000)081<0676:ROEVTS>2.3.CO;2

[20] Mills JN, Johnson JM, Ksiazek TG, Ellis BA, Rollin PE, Nichol ST, A survey of hantavirus reservoir populations in selected United States national parks. Am J Trop Med Hyg. 1998;58:525–32. 9574803 10.4269/ajtmh.1998.58.525

[21] Dizney LJ, Jones PD, Ruedas LA. Efficacy of three types of live traps used for surveying small mammals in the Pacific Northwest. Northwestern Naturalist. 2008;89:171–80. 10.1898/NWN08-18.1

[22] Parmenter RR, Yates TL, Anderson DR, Burnham KP, Dunnum JL, Franklin AB, Small-mammal density estimation: a field comparison of grid-based vs. web-based density estimators. Ecol Monogr. 2003;73:1–26. 10.1890/0012-9615(2003)073[0001:SMDEAF]2.0.CO;2

[23] O’Farrell MJ, Clark WA, Emmerson FH, Juarez SM, Kay FR, O’Farell TM, Use of mesh live trap for small mammals: are results from Sherman live traps deceptive? J Mammal. 1994;75:692–9. 10.2307/1382517

[24] Williams DF, Braun SE. Comparison of pitfall and conventional traps for sampling small mammal populations. J Wildl Manage. 1983;47:841–5. 10.2307/3808622

[25] Mills JN, Yates TL, Childs JE, Parmenter RR, Ksiazek TG, Rollin PE, Guidelines for working with rodents potentially infected with hantavirus. J Mammal. 1995;76:716–22. 10.2307/1382742

[26] Feldmann H, Sanchez A, Morzunov S, Spiropoulou CF, Rollin PE, Ksiazek TG. Utilization of autopsy RNA for the synthesis of the nucleocapsid antigen of a newly recognized virus associated with hantavirus pulmonary syndrome. Virus Res. 1993;30:351–67. 10.1016/0168-1702(93)90101-R 8109165

[27] Gannon WL, Sikes RS; Animal Care and Use Committee of the American Society of Mammalogists. Guidelines of the American Society of Mammalogists for the use of wild mammals in research. J Mammal. 2007;88:809–23. 10.1644/06-MAMM-F-185R1.1 PMC5909806 29692469

[28] Thomas L, Laake JL, Strindberg S, Marques FFC, Buckland ST, Borchers DL, Distance home page. St. Andrews (Scotland): University of St. Andrews, Research Unit for Wildlife Population Assessment; 1998 [cited 2008 Aug 16]. Available from http://www.ruwpa.st-and.ac.uk/distance

[29] Simpson EH. Measurement of diversity. Nature. 1949;163:688. 10.1038/163688a0

[30] Brower JE, Zar JH, von Ende CN. Field and laboratory techniques for general ecology. 4th ed. Dubuque (IA): William. C. Brown Publishers; 2002. p. 178–80.

[31] Dalgaard P. Introductory statistics with R. New York: Springer Science+Business Media, Inc.; 2002. p. 116.

[32] Crawley MJ. Statistical computing: an introduction to data analysis using S-Plus. West Sussex (UK): John Wiley and Sons, Ltd; 2002.

[33] Clarke KR, Warwick RM. Change in marine communities: an approach to statistical analysis and interpretation. 2nd ed. Plymouth (UK): Primer-E Ltd; 2001. p. 3–4.

[34] Keesing F, Holt RD, Ostfeld RS. Effects of species diversity on disease risk. Ecol Lett. 2006;9:485–98. 10.1111/j.1461-0248.2006.00885.x 16623733

[35] Ostfeld RS, Holt RD. Are predators good for your health? Evaluating evidence for top-down regulation of zoonotic disease reservoirs. Front Ecol Environ. 2004;2:13–20.

[36] Begon M. Effects of host diversity on disease dynamics. In: Ostfeld RS, Keesing F, Eviner VT, editors. Effects of ecosystems on disease and of disease on ecosystems. Princeton (NJ): Princeton University Press; 2008. p. 12−29.

[37] Merianos A. Surveillance and response to disease emergence. Curr Top Microbiol Immunol. 2007;315:477–509. 10.1007/978-3-540-70962-6_19 17848076 PMC7121157

[38] Daszak P, Cunningham AA, Hyatt AD. Emerging infectious diseases of wildlife–threats to biodiversity and human health. Science. 2000;287:443–9. 10.1126/science.287.5452.443 10642539

[39] Ezenwa VO, Godsey MS, King RJ, Guptill SC. Avian diversity and West Nile virus: testing associations between biodiversity and infectious disease risk. Proc R Soc Lond B Biol Sci. 2006;273:109–17. 10.1098/rspb.2005.3284 PMC1560012 16519242

[40] Daily GC, Ellison K. The new economy of nature: the quest to make nature profitable. Washington: Island Press; 2002.

